# Diverse and distributed haemodynamic effects of theta burst stimulation in the prefrontal cortex

**DOI:** 10.1016/j.ynirp.2025.100282

**Published:** 2025-08-27

**Authors:** Amy Miller, Melanie Burke

**Affiliations:** School of Psychology, Faculty of Medicine and Health, University of Leeds, LS2 9JT, UK

**Keywords:** cTBS, iTBS, Transcranial Magnetic Stimulation (TMS), Functional Near Infra-Red Spectroscopy (fNIRS), Non-invasive brain stimulation (NIBS), Dorsolateral Prefrontal Cortex (DLPFC)

## Abstract

**Background:**

Theta Burst Stimulation (TBS) is a form of non-invasive brain stimulation that can induce neuroplastic changes in the underlying intracortical areas. It has significant potential in clinical and research settings for modulating cognitive and motor performance. Little is known about how TBS affects oxygenations levels within and across brain hemispheres during stimulation of the Dorsolateral Prefrontal Cortex (DLPFC). This study aimed to investigate blood oxygenation levels in the DLPFC during TBS, using concurrent functional Near-Infrared Spectroscopy (fNIRS).

**Methods:**

44 young adults completed within-subjects 2 × 2 design with 4 conditions that included intermittent TBS (iTBS), and continuous TBS (cTBS) stimulation applied to the left and right DLPFC. FNIRS was recorded concurrently, with 12 optode channels spanning across the left, medial and right prefrontal cortex.

**Results:**

Findings focused on corrected significant effects that revealed clear neurovascular coupling during stimulation. Right hemisphere iTBS stimulation on the DLPFC resulted in excitation within and between hemispheres as expected, however left hemisphere stimulation decreased oxygenation levels both ipsilaterally and contralaterally. CTBS on the right and left hemisphere revealed reductions in HbO as expected in support of previous literature and potential LTD-like effects.

**Conclusion:**

This is the first study to show the extent and dispersion of blood-oxygenation changes in the ipsilateral and contralateral hemispheres during excitatory and inhibitory TBS applied to the DLPFC. The findings demonstrate that TMS stimulation may originate from more global and interhemispheric effects, but that iTBS on the left-DLPFC induces decreases in oxygenated haemoglobin (HbO) providing the potential links for beneficial effects in cognition.

## Highlights

• FNIRS provides an optimal method for monitoring concurrent brain stimulation.
• CTBS shows bilateral increases in deoxygenated blood (HbR) with stimulation in DLPFC.
• Left iTBS on DLPFC unexpectedly caused bilateral increases in HbR during stimulation.
• Right iTBS on DLPFC showed increases in HbO bilaterally during stimulation.

## 1 Introduction

Transcranial Magnetic Stimulation (TMS) is a non-invasive brain stimulation technique that works by alternating magnetic fields to induce depolarization or hyperpolarization in the underlying tissue via the transmission of electrical currents. TMS is a safe, reliable and well-established method for inducing neural activity and cognitive effects, used in both clinical and research settings. It can achieve neural firing patterns akin to long-term potentiation (LTP) and long-term depression (LTD) via repetitive stimulation at high (>5 Hz) or low (<1 Hz) frequencies ( Di Lazzaro et al., 2005). Huang and colleagues (2005) identified a stimulation protocol known as Theta Burst Stimulation (TBS) that modulates gamma oscillations via theta-gamma coupling in the brain associated with non-Hebbian plasticity (Cárdenas-Morales et al., 2010). A review by Wischenewski and Schutter (2015) of sixty-four studies showed increased motor evoked potentials lasting up to 60 min for intermittent TBS (iTBS) and 50 min for continuous TBS (cTBS), supporting TBS-induced cortical plasticity in the motor system (Chen and Udupa, 2009). Additionally, TBS is more efficient, with shorter durations of 40 or 190 s compared to traditional repetitive TMS (rTMS) protocols lasting up to 60 min (Chung et al., 2015).

There is good evidential support for TBS causing long-term plasticity in the underlying neurons using pharmacological, physiological, and behavioural measures post stimulation (Huang et al., 2007). Pharmacological studies have shown that the N-methyl-D-aspartate (NMDA) receptors are crucial for effective brain stimulation, as blocking these using Memantine, results in removing TBS effects (Huang et al., 2007). Physiologically, TBS has been shown to affect the I-waves (indirect waves) involved in pyramidal cell excitability and inhibitory interneuron connectivity vital for neuroplasticity in the motor cortex. Conversely, the D-waves (direct waves) that reflect direct excitation of the pyramidal neurons, are less affected indicating an effect that is beyond simple neuronal excitation (Ziemann et al., 1998). Interestingly, iTBS shows enhancement of the later I-wave (indirect wave) amplitude causing LTP-like effects, whereas cTBS shows reduction in the early I-wave resulting in LTD like effects (for review see Opie and Semmler, 2021).

Despite these advancements in understanding for the neural mechanisms behind TBS activity, the modulatory effects within and between hemispheres during TBS induction is currently limited, with the majority of previous studies focused on rTMS. To assess more regional related oxygenation level effects of TBS functional near-infrared spectroscopy (fNIRS) provides an optical neuroimaging modality that quantifies cortical hemodynamic responses by measuring changes in oxygenated and deoxygenated hemoglobin, with a spatial resolution of ∼1–3 cm and a sampling rate sufficient to capture rapid neurovascular dynamics. The novelty of this imaging modality is due to its non-magnetic components allowing concurrent TMS–fNIRS that can enable time-locked assessment of stimulation-induced cortical hemodynamic responses, providing mechanistic insights into neurovascular coupling and cortical excitability that extend beyond those obtainable from offline or post-hoc measurement paradigms.

In addition, many of these previous studies focus on the motor cortex to assess induced plasticity changes, which may not be representative of other cortical regions. One region of particular interest in the literature, in relation to neuroplasticity is the dorsolateral prefrontal cortex (DLPFC), due to its clear relationship with cognition and executive functioning (Di Lazzaro et al., 2021) and the potential to improve cognitive outcomes in those with cognitive impairments (Miller et al., 2023). A number of previous studies have used concurrent TMS-fNIRS in the DLPFC and a systematic review by Curtin et al. (2019) identified 10/53 papers looking at DLPFC activity during rest, with only one study investigating iTBS and no studies on cTBS effects. A more recent review identified 13 papers that also utilized concurrent TMS and fNIRS in the DLPFC (Xia et al., 2024). This review identified that high frequency rTMS most consistently produced facilitatory excitation effects, with large variability in the reporting and protocols used. From the 13 studies that used fNIRS and TMS, none of these studies examined TBS protocols on the DLPFC. Since these reviews, two papers have used concurrent fNIRS and iTBS stimulation on the left DLPFC. One investigated gender differences in HbO finding higher oxygenated haemoglobin (HbO) in males compared to females under the stimulation site (Kan et al., 2024). The other study by Xia and colleagues (2025) found increased deoxygenated hemoglobin (HbR) during left DLPFC iTBS stimulation using fNIRS. Further conflict in the literature has been identified using concurrent iTBS on the left DLPFC and concurrent fMRI with findings showing increased BOLD activity bilaterally (Chang et al., 2024).

These previous findings show clear diversity in the effects reported during iTBS in the DLPFC with no clear consensus and also highlights the known inter and intra individual variability in response to brain stimulation. The current study aims to address these identified disparities by using a within subject double dissociation design to compare ipsilateral and contralateral effects of left and right DLPFC stimulation using both iTBS and cTBS protocols. Using this 2 × 2 design we aimed to assess differences in oxygenation and deoxygenation levels produced by excitatory (iTBS) and inhibitory (cTBS) protocols in the DLPFC during stimulation, using concurrent fNIRS. This multi-modal approach will provide novel insights into the immediate neural effects of these stimulation techniques. Building on prior research, we hypothesize that intermittent theta burst stimulation (iTBS) will elevate oxygenation levels in the underlying cortex during stimulation, when using an event-related design. The effects of iTBS on the contralateral hemisphere are less predictable and have not been previously documented. Additionally, we anticipate that continuous theta burst stimulation (cTBS) will decrease blood oxygenation levels at the stimulation site in the prefrontal cortex during the 40s stimulation, with some inhibitory effects transmitted to the opposite hemisphere.

## 2 Methods

### 2.1 Participants

44 participants, aged 18–25 years old (M = 20.18, SD = 1.32) with 26 identifying as female and 18 as male were recruited for the study. Participants had no known neurological, developmental or psychological deficits and had normal or corrected to normal vision. Participants were right-handed and monolingual. Prior to taking part, participants were provided with an information sheet, completed a medical history questionnaire, and provided their written informed consent. The study was approved by the University of Leeds Ethics Committee on December 30, 2022 (PSYC-698), and fully abided by the British Psychological Society Code of Human Research Ethics (Oates et al., 2021), as well as the Declaration of Helsinki (World Medical Association. World Medical Association, 2013).

### 2.2 Study design

**Fig. 1:**
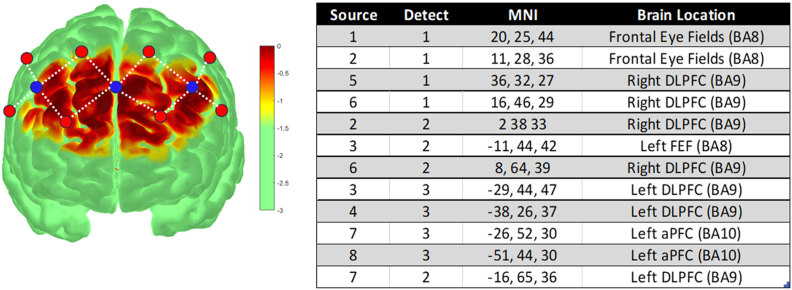
The brain image (left) shows the approximate location of the optodes and channels on a template brain (Colin 27) using averaged data from all participants. 12 channels were created from 4 unsplit and 8 split sources (red dots), and 3 detector optodes (blue dots). The sensitivity profile shows the spatial distribution of activity across the prefrontal cortex indicating good and even coverage of measured activation. The table (right) shows the estimated anatomical location (and Brodmann area) of the midpoints of each source-detector channel in Montreal Neurological Institute (MNI) coordinates. Images and table created using Atlas Viewer (v2.44.0, R2017b). Dorsolateral prefrontal cortex (DLPFC), frontal eye fields (FEF), anterior prefrontal cortex (aPFC).

The location of the stimulation was focused over the Dorsolateral Prefrontal Cortex (DLPFC) in the left and right hemisphere corresponding to the EEG 10–20 configuration of F3 and F4 respectively and was allocated to Brodmann area 9 (see Fig. 1). The study utilized a 2 × 2 repeated measures design, within subject crossover in which stimulation type (cTBS/iTBS) and cerebral hemisphere (left/right) were the independent variables resulting in 4 conditions: left hemisphere cTBS (LH-cTBS), right hemisphere cTBS (RH-cTBS), LH- iTBS, RH iTBS. Each participant performed all 4 conditions in a random order in separate sessions spaced 1 week apart. Blood oxygenation level dependent (BOLD) responses as measured by fNIRS and HbO response to stimulation was the dependent variable. 12 channels were positioned over the orbitofrontal cortex and dorsolateral prefrontal cortex as shown in Fig. 1 (table). Sensitivity profiles were calculated using AtlasViewer (Huppert et al., 2009; see Fig. 1) using Monte Carlo procedure for the group, and projected coordinates of each optode (Fig. 1). TBS stimulation was placed over detector optodes 1 (F4) and 3 (F3) on each subject.

### 2.3 TMS protocol

TMS was administered using a Magstim Rapid^2^ (Magstim Company Ltd). Depending on the experimental condition, either cTBS or iTBS was administered with a figure of 8 coil at an optimal 45° angle to the cortex (Thomson et al., 2013). cTBS stimulation consisted of administering triplets of 50 Hz electromagnetic pulses for 200ms uninterrupted at 50 % power for 40 s, with total stimulation consisting of 600 pulses. Alternatively, iTBS stimulation consisted of administering triplets of 50 Hz electromagnetic pulses every 200ms at 50 % of the TMS machine power output, repeated every 10 s for 190 s, with total stimulation consisting of 600 pulses (Huang et al., 2005; Pabst et al., 2022).

The TMS coil was held perpendicular to the scalp, with the centre of the coil above the region of interest; this was area F3 of the LH, corresponding to the left DLPFC, or area F4 of the RH, corresponding to the right DLPFC (Herwig et al., 2003). The International 10–20 EEG was used to identify the DLPFC using F3 and F4 when the cap was placed over CZ using raison and inion measurements (Herwig et al., 2003). The hemisphere of stimulation was dependent on the experimental condition. An automatic trigger was sent from the TMS system to the fNIRS system that was used to align fNIRS data to TMS onset and offset.

TMS is generally well-tolerated and painless for most participants (Najib and Horvath, 2014; Rossi et al., 2021). There are well established safety protocols for TMS which were followed during this study, and TMS is safe when used in accordance with these protocols (Rossi et al., 2021). No participant had a counterindication to TMS during this study (Rossi et al., 2021).

### 2.4 Functional near-infrared spectroscopy

A 12 channel Oxymon Mk II fNIRS system (Artinis Medical Systems) was used to measure changes in the concentration of oxygenated blood (HbO), deoxygenated blood (HbR) and total change in oxygenation (tHb = HbO-HbR) in μmol/L. Data was collected at a frequency rate of 100 Hz, and the differential path factor (DPF) was adjusted for age related differences in the brain using the formula: DPF = 4.99 + 0.067∗(age^0.814) (Duncan et al., 1996). The optodes were attached to a black neoprene head cap that absorbed external light and were organised symmetrically over the DLPFC of both hemispheres, with approximately 30 mm distance between the source and detector optodes resulting in a penetration depth of around 8 mm. The optode template design comprised 3 detector and 8 receiver optodes (see Fig. 1) resulting in 8 split and 4 unsplit channels spanning the left, midline and right prefrontal cortex. To reduce movement artefacts, participants were asked to remain as still as possible, the cap was secured using an elastic chin strap to maintain optimal optode-scalp contact, and all fNIRS cables were attached to a drip stand for stability. During stimulation, real time concentration changes in HbO, HbR and total Haemoglobin change (tHb) were displayed using Oxysoft (Version 3.0.103.3) (Artinis Medical Systems, n.d.).

FNIRS is non-invasive, safe, portable, and tolerant to movement artefacts (Irani et al., 2007; Pinti et al., 2020). Furthermore, it does not utilise electrical or magnetic signals, and therefore does not interact with the electromagnetic field generated by the TMS coil (Tian et al., 2012). By recording both oxygenation and deoxygenation levels can also provide confidence on the signal being derived from neurovascular coupling mechanisms. One limitation of the design is the increased distance of the TMS coil from the scalp of ∼12 mm due to the optode profile. Previous studies have identified a stimulator output of 30 % of the stimulator output over the DLPFC produces effective and consistent behavioural/cognitive effects (Miller et al., 2023; Burke and Coats, 2016; Burke et al., 2013). We therefore adjusted the stimulator output for the increased TMS coil to scalp distance by using the equation Adjusted Output = 30+(2∗(12)) = 54, and then rounded this to 50 % of stimulator output as suggested by Cukic et al. (2009) that is also in-line with Stokes et al. (2005).

### 2.5 Data analysis

All data files were converted into a ∗.NIRS format using the oxysoft2matlab function (MatLab R2022a, Mathworks Inc) and stored in folders according to the Brain Imaging Data Structure (BIDS) framework. Functional NIRS data is subject to biological and technical artefacts and so prior to pre-processing the QTNirs toolbox was used to identify suboptimal channels for each participant. Channels that didn't reach a cut-off of 70 % signal quality during baseline and TBS recording were excluded from further analysis. In addition, any participant who had <50 % suboptimal channels were excluded from the subsequence analysis. This resulted in 4 participants being excluded for the cTBS condition, resulting in 40 participants, whereas for iTBS all participants achieved this criterion resulting in all 44 participants included in further analysis (Santosa et al., 2018).

Data was then subjected to a standard pre-processing pipeline within the NIRS toolbox administered in the MatLab environment (Bradley et al., 2022; Santosa et al., 2018). A bandpass filter (0.001–0.25 Hz) removed psychological noise before data was converted from haemodynamic intensity raw data into optical density (OD) using the modified Beer-Lambert Law. A GLM model using the FIR basis function to model the TBS data. Using the onset event triggers generated from the TMS machine to the fNIRS input we could accurately align fNIRS data with TBS onset. For iTBS, the stimulation matrix comprised 20 × 10s epochs that were averaged within participants, before generating a group level HbO and HbR. For cTBS, an average change from baseline to the 40s stimulation time was calculated for each participant before group level HbO and HbR were generated. Changes in HbO and HbR from baseline were calculated independently for each of the 12 channels to each of the 4 conditions: LH iTBS, RH iTBS, LH cTBS and RH cTBS. These group level results were followed by T-tests to identify significant effects.

**Fig. 2:**
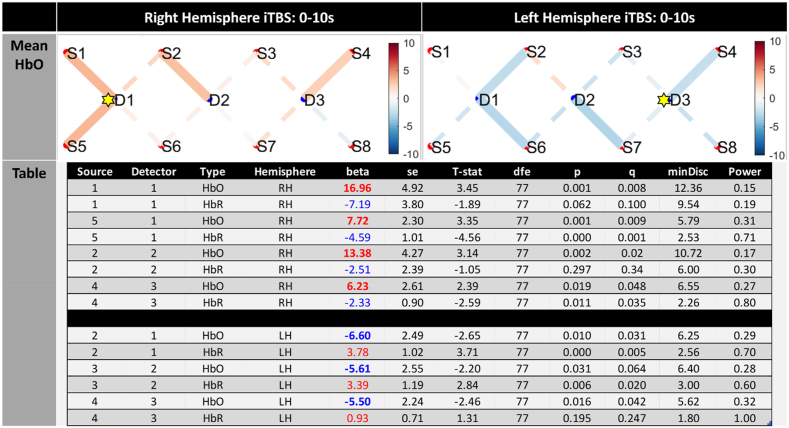
Oxygenation level changes for all 44 subjects during iTBS on the right DLPFC (D1 aka F4) and left DLPFC (D3 aka F3). The iTBS stimulation is averaged over the 2 s of stimulation followed by 8 s delay over the 20 epochs are shown in the upper 2 graphs (mean HbO). Statistical results for channels that show significant differences (at FWE q < 0.05 level) from baseline, alongside clear neurovascular coupling is included in the table presented at the bottom of the figure. The table shows significant channel activation for HbO channels reaching p < 0.05, where q denotes family-wise correction (FWE). Both oxygenated (HbO) and deoxygenation (HbR) significance levels are reported from baseline, alongside conditions, standard error (se), T-stat, degrees of freedom (dfe), minimum discoverable effects (minDisc), and power. Positive increases in activity are in red and decreases shown in blue with T-stat levels indicated by intensity of colour. The Hemisphere column highlights whether iTBS was on the left hemisphere (LH = left DLPFC) or on the right hemisphere (RH = right DLPFC) The yellow star denotes location of iTBS stimulation.

**Fig. 3:**
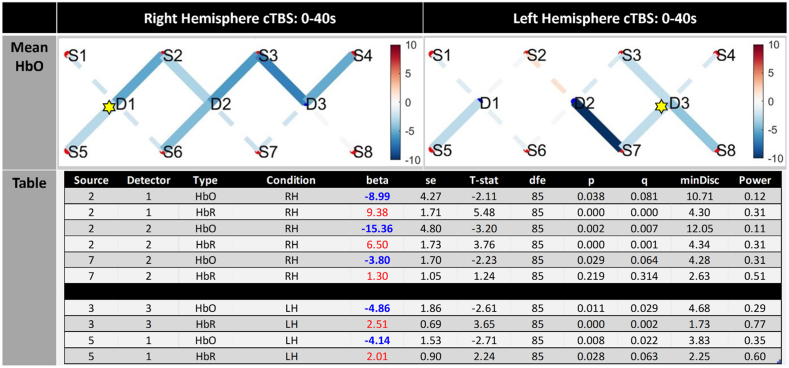
The mean oxygenation level changes for all 40 subjects during cTBS on the right DLPFC (D1 aka F4) and left DLPFC (D3 aka F3) is shown in the upper 2 images. The cTBS stimulation is averaged over the 40 s of stimulation for each participant and the intensity of the colour red (increased HbO) or blue (decreased HbO) reflects the level of change in oxygenation levels from baseline. Statistical effects for channels that show significant differences (at FWE q < 0.05 level) from baseline, alongside clear neurovascular coupling (i.e. associated decrease in HbR) are included in the table presented at the bottom of the figure. The table shows significant channel activation for channels reaching p < 0.05, where q denotes family-wise correction (FWE). Both oxygenated (HbO) and deoxygenation (HbR′) significance levels are reported from baseline, alongside conditions, standard error (se), T-stat, degrees of freedom (dfe), minimum discoverable effects (minDisc), and power. The Hemisphere column highlights whether iTBS was on the left hemisphere DLPFC (LH) or on the right hemisphere (RH). The yellow star denotes location of cTBS stimulation.

The resultant data shown below was subjected to Bonferroni correction (family-wise error correction) for multiple comparisons as shown by the reported q value in Fig. 2, Fig. 3. In addition, to ensure our data was neuronally driven and not noise related, an additional level of control was performed on the results data whereby significant changes in oxyhaemoglobin (HbO) was only selected if there was a corresponding negative correlation with deoxyhaemoglobin (HbR) providing confidence that the data was showing clear neurovascular coupling in the response (Kinder et al., 2022).

## 3 Results

### 3.1 iTBS effects in the DLPFC

To evaluate how the haemodynamic response (HDR) was affected by iTBS stimulation we plotted filtered GLM modelled HbO block averages from all participants for the left hemisphere (LH) and right hemisphere (RH) (see supplementary material). To evaluate mean changes in oxygenated blood during iTBS we modelled 10 s epochs that included 2s stimulation followed by an 8 s rest (see Fig. 2). The Mean HbO images show channels of activity that reach FWE(q) < 0.05 level (Fig. 2, table). The table at the bottom of the image presents only significant channels where increases in HbO was coupled with increases in HbR i.e. evidence of neurovascular coupling. For ***right hemisphere iTBS*** results show significant positive increases in HbO near the site of stimulation [S1/D1, S5/D1], in posterior midline PFC [S2/D2] and contralaterally in left DLPFC (BA9) suggesting an influx of oxygenated blood into these areas during the 10 s iTBS stimulation epochs. From these S5/D1 and S4/D3 revealed corresponding increases in deoxygenated blood (HbR) demonstrating clear neurovascular coupling. ***Left hemisphere iTBS*** revealed some widespread decreases in oxygenated blood (HbO) ipsilaterally [S4/D3], in the midline [S7/D2] and contralaterally [S6/D1, D2/D1] to the site of stimulation. From these significant effects (at FWE <0.05 level) only 2 channels revealed corresponding decreases in HbR [S2/D1, S3/D2].

### 3.2 Effects of cTBS in the DLPFC

The mean haemodynamic response (HDR) over the 40 s epoch in continuous theta burst stimulation for all 40 participants is provided in the supplementary materials (‘HDR’). The mean changes in oxygenation levels during cTBS are shown in Fig. 3. We found significant decreases in HbO in 5 channels principally located around the site of stimulation during ***left hemisphere cTBS*** in S8/D3, S3/D3, S7/D3, one in the midline S7/D2, and one contralateral to the site of stimulation [S5/D1] in right DLPFC. Only 2 of these channels revealed corresponding increases in deoxygenated blood (HbR) indicative of neurovascular coupling in ipsilateral and contralateral DLPFC respectively. ***Right hemisphere cTBS*** on the DLPFC revealed widespread significant decreases in oxygenation levels in 7 channels across the prefrontal cortex. This deactivation shown at the FWE (q) < 0.05 level was present on ipsilateral, midline and contralateral prefrontal areas. However, on closer inspection of the data and when only including activity that revealed a positive increase in HbR only 2 channels [S2/D1 and S2/D2] in the ipsilateral (right) hemisphere were identified (see Fig. 3, table).

Finally, to confirm and strengthen our interpretation of the specific TBS and hemisphere effects a 2 (iTBS or cTBS) x 2 (left or right-hemisphere) within subject repeated-measures ANOVA was conducted on beta weights and results confirm a significant main effect of TBS protocol F_(1,11)_ = 20.5, p < 0.001 and a significant TBS × hemisphere interaction F_(1,11)_ = 12.2, p = 0.005, but with no main effect of hemisphere p = 0.06. This confirms our interpretation that the 2 TBS protocols are inducing differing effects in the brain, and that these effects are hemisphere dependent.

## 4 Discussion

The aim of this study was to utilise concurrent TMS and fNIRS to measure changes in oxygenated and deoxygenated blood across the prefrontal cortex during iTBS and cTBS on the left and right DLPFC in participants with no known neuropsychiatric disorders. This 2 × 2 within-subject (repeated-measures) factorial design provides novel insights into the effects of theta burst stimulation (TBS) both ipsilaterally and contralaterally during stimulation. This is the first study to compare left (F3) versus right (F4) hemisphere DLPFC stimulation in both iTBS and cTBS protocols at the site of stimulation (ipsilateral), in the midline, and on the contralateral PFC enabling a comprehensive assessment of cortical oxygenation changes.

### 4.1 iTBS effects

To summarize the findings, we found iTBS on the right DLPFC (F4) resulted in increased oxygenation levels (HbO) in both ipsilateral and contralateral BA9. This is the first study to show excitation ipsilateral to the site of stimulation in iTBS. These effects are also aligned with the mean haemodynamic response profiles found in this study, and previous literature on iTBS effects (for review see Xia et al., 2024). Two previous papers have reviewed instantaneous effects of high frequency TMS applied to the right hemisphere in the DLPFC, and both found increased activity contralaterally on the left PFC in-line with findings reported here (Aoyama et al., 2009; Hanaoka et al., 2007). In addition, a previous study reported increased activity in healthy controls during right DLPFC stimulation (Ćurčić-Blake et al., 2022), but only recorded fNIRS from the inferior parietal lobe suggesting increased activity might be more widespread than the PFC.

Intermittent TBS on the left DLPFC (F3) induced the opposite effect showing a reduction in HbO and subsequent increase in HbR during stimulation in ipsilateral, midline and contralateral PFC. Our study is consistent with a recent study by Xia et al. (2025) who also identified clear increases in HbR and corresponding decreases in HbO to iTBS stimulation using a similar methodology. These findings are contrary to much of the previous literature using high frequency TMS on the left DLPFC, that has found increases in oxygenation levels ipsilaterally (Curtin et al., 2017; Cao et al., 2013). For example, Curtin et al. (2017) applied iTBS to the left DLPFC with concurrent fNIRS and found increased HbO locally at the site of stimulation, but did not report any contralateral effects.

Many of the studies included in TMS reviews stimulate the left-DLFPC but provide little justification for targeting this hemisphere. The current study sheds light on why left DLPFC stimulation specifically may provide therapeutic effects. A prolonged reduction in HbO during stimulation, may optimize and balance bilateral neural activity, especially in older populations, due to hyperactivity issues that results in more optimal cognitive effects post-stimulation. There are several potential reasons for why left hemisphere iTBS over the DLPFC may cause a reduction in HbO during stimulation. Firstly, it is worth noting that left and right DLPFC are functionally interconnected through transcallosal inhibitory pathways. Excitatory iTBS at F3 may have strengthened inhibitory projections to the right DLPFC, leading to a suppression of activity in the right hemisphere, which could explain reduced HbO bilaterally. In support of this, Pulopulos et al., (2022) identified the left DLPFC as inducing inhibitory proactive control by top-down regulatory mechanisms that prevent inappropriate responses and anticipate cognitive control, while right DLPFC was more involved in reactive bottom-up attentional control.

Another possible explanation of reduced HbO during left DLPFC iTBS at rest could be that we were activating the Default Mode Network (DMN). The DMN often works to reduce activity in cognitive demanding regions, and therefore activating left DLPFC could result in a net reduction in HbO. This net reduction in DMN is associated with enhanced focus, improved problem solving and better cognitive control (Fox et al., 2005). The reduced activity in the DMN is also thought to provide benefit in mood (for clinical guidelines see Lefaucheur et al., 2020) and cognitive outcomes for those with cognitive impairments (Miller et al., 2023). It is thought that left DLPFC is beneficial for depression due to the presumed up-regulation of the left hemisphere to reduce hemispheric imbalance (Kimbrell et al., 1999). Our data suggests that therapeutic benefit could infact be due to distal down-regulation of the right DLPFC during left hemisphere stimulation that is producing the effect and argues for a more individualized approach to stimulation (Luber et al., 2017). Furthermore, evidence from the Cognitive Control Network (CNN) that is responsible for attention regulation and cognitive flexibility may also show decreased HbO due to functional optimization and neural efficiency across this network (Neubauer and Fink, 2009). In-line with our findings, Schulter and Colleagues (2018) also found decreased functional connectivity within the salience network involving PFC after left DLPFC excitatory rTMS, and the opposite (increased functional activity) with right DLPFC stimulation.

This diversity in functional effect of iTBS on the DPFC dependent on hemisphere is not entirely unexpected due to a plethora of literature acknowledging differences in left and right DLPFC activity during cognitive tasks in response to TMS (Xia et al., 2024). However, our results provide a novel contribution by being the first to demonstrating distinct effects of iTBS stimulation on the left and right DLPFC utilizing the same stimulation thresholds across all participants in a 2 × 2 within-subjects design, supporting different modes of action in the neurovascular response.

### 4.2 cTBS effects

Stimulation using continuous TBS in the DLPFC produced decreased HbO when applied over both left and right hemispheres. The right cTBS produced the more significant changes in HbO from baseline during stimulation specifically at the site of stimulation (D1/F4) and in the midline optodes related to dorsomedial prefrontal cortex. Left sided cTBS stimulation had significantly reduced HbO under the site of stimulation, and contralaterally in right DLPFC. This decreased in neural activity over the 40 s stimulation period was expected due to the known LTD-like effects proposed by Huang et al. (2005). Despite this, there is a plethora of literature that shows different effects with low-frequency repetitive TMS over left DLPFC, often resulting in increased activity in both local and remote brain regions (Nahas et al., 2001) or finding no significant effects (Caparelli et al., 2022). None of these previous studies in the DLPFC have shown decreased activity in local and remote brain areas during cTBS stimulation and so this is the first to demonstrate this effect using fNIRS.

Theta burst stimulation (5 Hz frequency of pulses) is thought to create LTD-like effects via a process of synaptic plasticity that are particularly prominent in the prefrontal cortex and hippocampus by influencing the intracellular calcium levels. For local effects under the stimulation site a moderate and sustained calcium influx activates a signaling cascades that results in LTD observed as decreased excitability and HbO levels in the PFC. It achieves this by increasing GABA release (inhibitory) and then also reducing excitatory responses by decreasing the amount and sensitivity of post-synaptic AMPA (alpha-amino-3-hydroxyl-5-methyl-4-isoxazolepropionic acid) receptors to glutamate. This shifts the balance that results in prolonged inhibition or suppression of cortical activity within the DLPFC (Hoogendam et al., 2010). As noted with iTBS above, the effects of stimulation can spread and disperse beyond the site of stimulation, and we show that it spreads to both midline and contralateral brain regions during the stimulation phase of the left and right DLPFC. Inhibitory networks associated with the DLPFC include the frontoparietal control network (FCN) and the default mode network (DMN, mentioned above). Therefore, demonstrating clear inhibitory influence in these networks that potentially result in the observed behavioral adaptations. Interestingly the FCN shows a right hemisphere PFC dominance in inhibitory control of the limbic system needed for emotion regulation (Grimshaw and Carmel, 2014) and cognition in terms of attentional control (Dodds et al., 2011). In support of this, we found that cTBS on the right DLPFC produced a significantly greater decreases in HbO (LTD-like effects) under the site of stimulation, than cTBS stimulation on the left hemisphere. This may also be attributed to greater sensitivity to dopamine and noradrenaline effects in right DLPFC as shown in studies investigating attention deficit hyperactivity disorders (Arnsten, 2009). The greater levels of inhibitory activity in midline and contralateral hemispheres in right cTBS also supports the right hemisphere having stronger network-level connections possibly providing greater potential for inhibitory control (Hausman et al., 2022).

It's worth noting that researchers have demonstrated intra- and inter-individual variability to TMS stimulation. This variance is thought to arise from several sources including: (i) local state-dependent effects whereby baseline brain oscillations and activity within the stimulated region might shape how the brain responds to stimulation (Bradley et al., 2022), (ii) remote network activity effects (Sack et al., 2024), and (iii) individual differences in anatomy and physiology of the PFC (Guerra et al., 2020).

### 4.3 Strengths and limitations

We are the first study to directly compare left versus right hemisphere effects of TBS using 2 (left/right hemisphere) x 2 (iTBS/cTBS) within subject design. The larger sample size used in this study allowed for very stringent measures of data pruning, alongside only reporting Bonferroni-corrected significant effects with confirmed neurovascular coupling (via HbO/HbR validation) a measure often missed in previous literature (Kinder et al., 2022). Significant HbO changes were observed in multiple channels, and the majority showed concurrent HbR changes consistent with canonical neurovascular coupling (i.e., increased HbO with decreased HbR). However, partial coupling (when HbO and HbR are not aligned) is not uncommon in fNIRS studies of the prefrontal cortex and may reflect a combination of factors, including inter-individual variability in cortical vasculature, regional differences in baseline oxygenation, and variability in cap placement relative to underlying gyri and sulci. In our study these channels were excluded based on predefined criteria to reduce the risk of misattributing systemic or artifactual signals to true neural activity. Future studies incorporating short-separation channels or physiological monitoring (e.g., heart rate, respiration) could also help further disentangle systemic influences from true cortical responses.

In addition, another important consideration is that previous studies looking at iTBS and fNIRS have used block designs for averaging effects over the full stimulation period. Our approach was to use an event-related design for modelling the data as we acquired accurate time-synced markers for the 2 s stimulation followed by the 8 s rest. This has provided novel and valuable insights into the immediate hemodynamic effects both under the coil and across the more dispersed PFC network. It should be noted that no sham was needed in this study given that we were not measuring behavioral/cognitive outcomes (removing psychological effects). Auditory and somatosensory effects were also consistent across all participants within conditions and given the prefrontal cortex is not a sensory specific region we would not predict these would have influenced our results.

One potential limitation could be the choice to utilise a consistent and single stimulation threshold for all participants in this study instead of using individuals resting motor threshold. This decision was based on 3 key lines of reasoning: (i) motor cortex and DLPFC have different excitability profiles (Boroojerdi et al., 2002; Tik et al., 2023) and so utilizing an arbitrary value induced on the motor cortex could have no relation to activity in the DLPFC and could over or under stimulate this area; (ii) to avoid variability in the intensity related effects that are non-linear in the DLPFC (Zhang et al., 2022), then a fixed output would provide the most comparable results across our population; (iii) to allow for understanding network-wide effects across the DLPFC and not just local effects we feel consistency in stimulation levels provides uniformity in these effects and enhances the potential reproducibility of outcomes. An additional potential limitation is due to the method of localization of left and right DLPFC. In this study we used anatomical localization based on the 10-20-system of EEG (Amassian et al., 1989). A potentially better approach would be to use functional brain images alongside neuronavigation technology to identify the specific regional response within an individual to a given cognitive task. One final limitation is that we did not report effects post-stimulation that are also important to consider when using non-invasive brain stimulation, however these effects were beyond the scope of this study.

## 5 Conclusions

To our knowledge, no previous study has reported the ipsilateral and contralateral effects of intermittent and continuous TBS when comparing left and right DLPFC stimulation, using a within subject design. Our novel findings demonstrate a clear asymmetry of effects of iTBS between hemispheres in the DLPFC. Right-sided iTBS induces LTP-like effects during the stimulation phase, but left iTBS on the DLPFC results in LTD-like effects. This finding supports different modes of action for cognitive control via possible inhibitory ‘top-down’ mechanisms in the left DLPFC, and more reactive control in the right DLPFC during the stimulation. Continuous TBS resulted in expected LTD-like effects across the whole of the PFC. Furthermore, higher shifts in inhibitory effects were observed in the right cTBS indicating an optimal site for activating inhibitory networks. We recommend that future studies carefully select their stimulation protocols (cTBS or iTBS) and hemisphere of stimulation in the PFC in accordance with potential network-effects in mind.

## CRediT authorship contribution statement

**Amy Miller:** Writing – original draft, Visualization, Validation, Software, Project administration, Methodology, Investigation, Formal analysis, Data curation. **Melanie Burke:** Writing – review & editing, Supervision, Software, Project administration, Methodology, Investigation, Funding acquisition, Formal analysis, Data curation, Conceptualization.

## Funding

The authors received support for the research and publication of this article from 10.13039/100014013UKRI IAA.

## Declaration of competing interest

The authors declare that they have no financial, personal, or professional conflicts of interest related to this work. No conflicts related to funding, patents, or other sources of financial support were associated with the preparation of this manuscript.

## Data Availability

Data will be made available on request.
