# Supplementary material for: Diverse and distributed haemodynamic effects of theta burst stimulation in the prefrontal cortex
Source: Neuroimage Rep. 2025 Aug 27;5(3):100282. doi: 10.1016/j.ynirp.2025.100282 (PMC12409971; doi:10.1016/j.ynirp.2025.100282)
Supplement: Multimedia component 1 [file mmc1.docx]

Supplementary Material

## Supplementary Figures


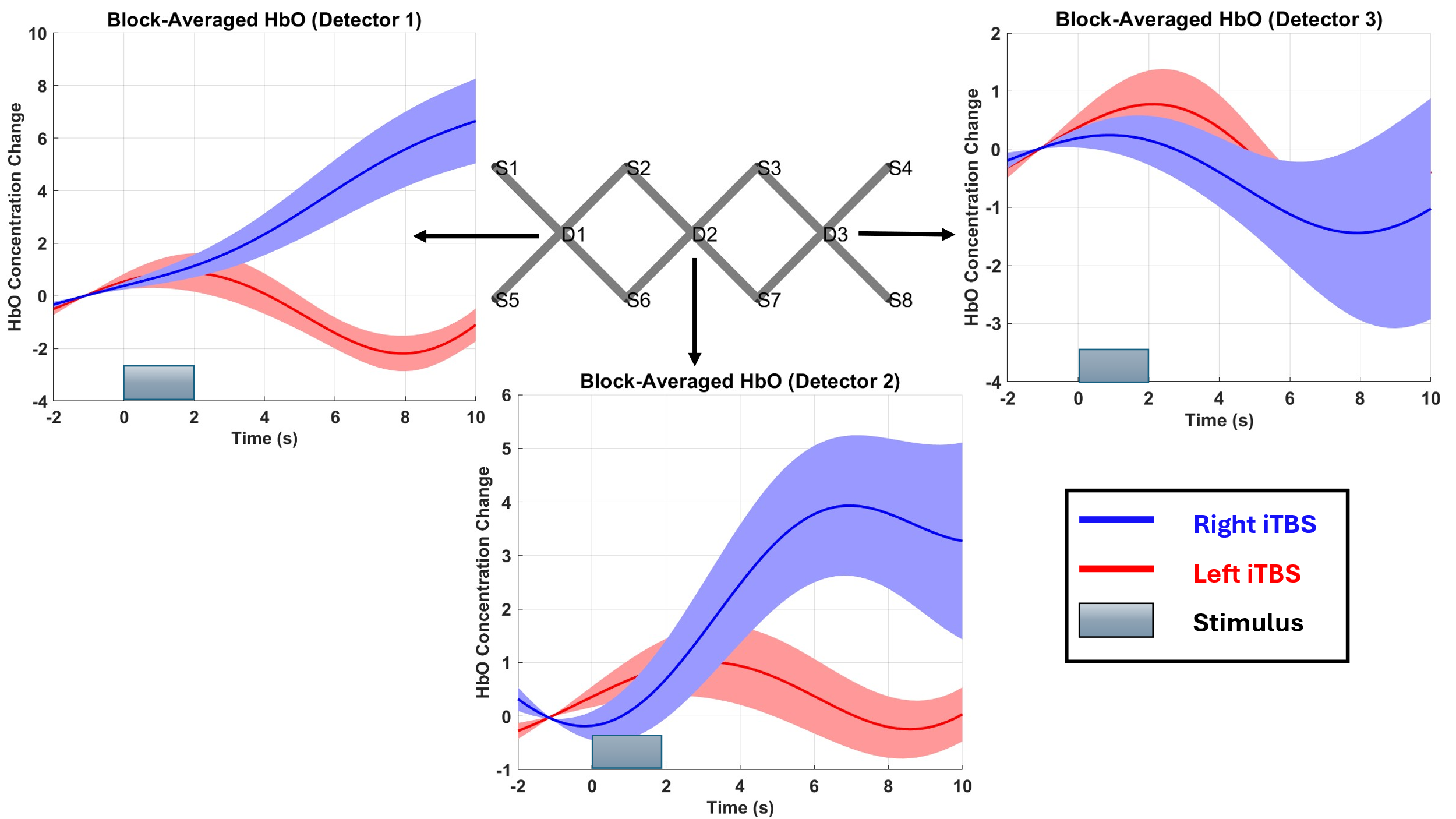


**Supplementary Figure 1:** The HbO concentration change plots for the iTBS protocol is shown with 2 seconds of stimulation shown by the grey bar, followed by 8 seconds of rest. These are the averaged profiles for all participants for right hemisphere iTBS (shown as a blue line) and stimulation of the left DLPFC shown as a red line. The blue and red edging around the central line represents the standard deviations of the 45 participants. The upper left graph shown averaged responses of 4 channels surrounding detector 1 positioned on the right side of the brain. The middle graph shows the averaged HbO profile of the four 4 channels surrounding the central detector 2. The upper right graph is the profile centred around detector 3 on the left side of the brain.


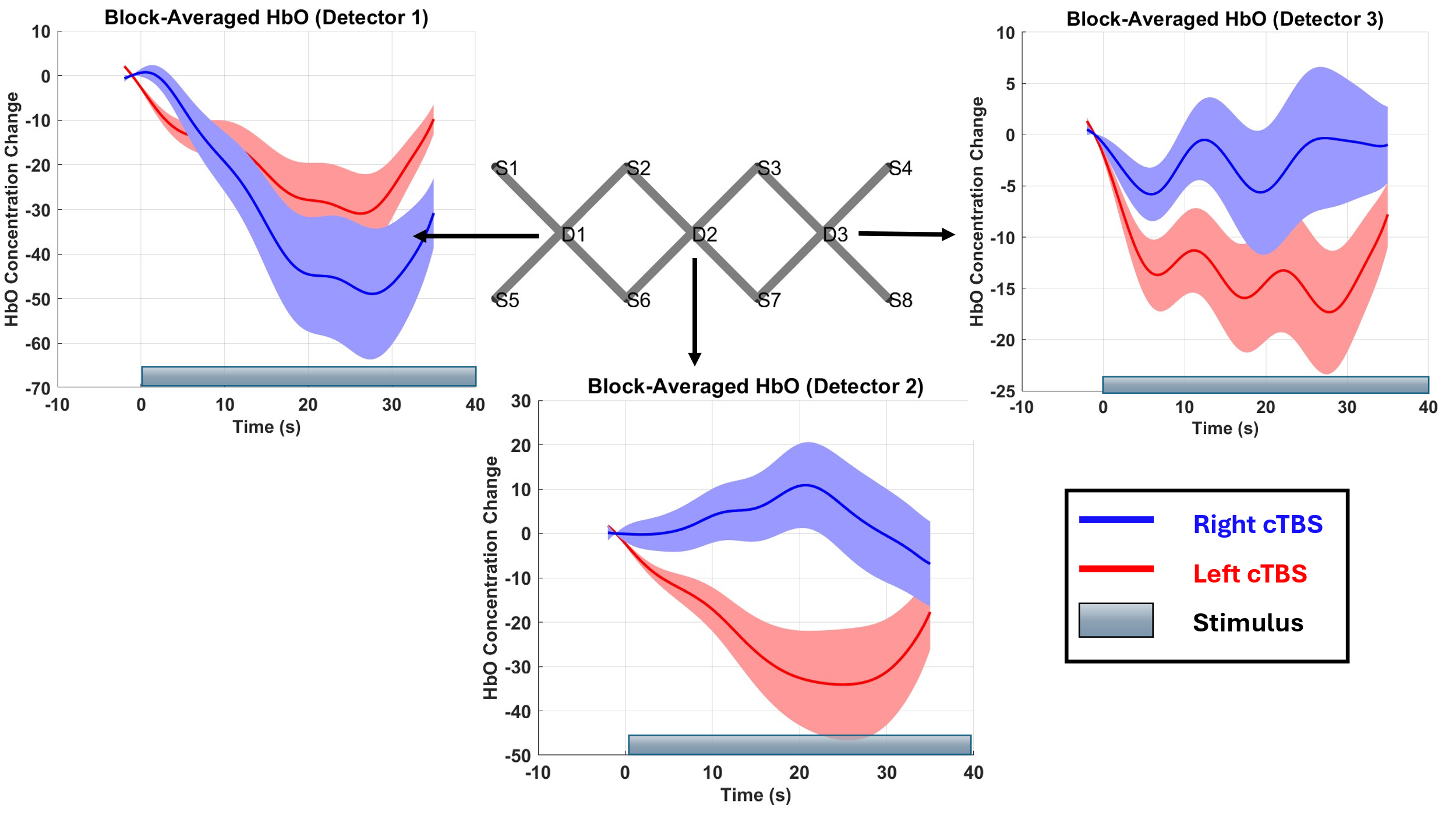


**Supplementary Figure 2:** This image shows HbO concentration change profiles from all participants to left (red line) and right (blue line) cTBS stimulation. In this protocol the stimulation was continuous for a 40 second period as shown by the grey bar at the bottom of the screen. The layout and detector clusters are the same as for Figure 1 above.
